# A study on the effects of regional differences on agricultural water resource utilization efficiency using super-efficiency SBM model

**DOI:** 10.1038/s41598-021-89293-2

**Published:** 2021-05-11

**Authors:** Yujie Huang, Xianke Huang, Munan Xie, Wei Cheng, Qin Shu

**Affiliations:** 1 College of Economics and Trade, Xinjiang Agricultural University, Urumqi, 830052 Xinjiang China; 2 Graduate SchoolChinese Academy of Social Sciences, Beijing, 00102 China; 3 University of North Carolina at Chapel Hill, Chapel Hill, NC USA

**Keywords:** Ecology, Environmental sciences, Environmental social sciences, Hydrology

## Abstract

This study evaluated the water resource utilization efficiency and resource consumption for planting, forestry, animal husbandry, and fishery in various regions of China. Using the super-efficiency Slacks-Based Measure (SBM) analysis method, the main agricultural pollution emissions (Chemical Oxygen Demand, ammonia nitrogen, and agricultural carbon emissions) were proposed as environmental constraints for the first time. The threshold regression model was used to measure the impact of agricultural water use efficiency on agricultural water consumption by constructing seven different explanatory variables. The results show that the overall utilization efficiency of agricultural water resources in China presents a fluctuating downward trend, and the regional differences are significant. A single threshold effect on agricultural water consumption was found in five variables: per capita water resources, disposable income, dependence on foreign trade, industrial structure, and Gross Domestic Product. The increase in each parameter will have a positive effect on agricultural water consumption. The relationship between agricultural water use efficiency and agricultural water use was non-linear when the government's attention and the rural labor force were used as threshold variables.

## Introduction

Water is an indispensable and irreplaceable natural resource, crucial to all life and the socio-economic development of society. At the global level, people do not fully understand the impact and pressure of daily activities on water resources, leading to excessive consumption and poor water resource management. The rapid increase in competitive water demand is a serious threat to water supply, especially in rapidly developing economies, and could endanger the well-being of billions of people.

At present, China is constantly promoting the transformation of its economic and social structure, which has accelerated its urbanization and industrialization. With the continuous growth in population, industrial and agricultural production, and urbanization, the limited water resources and water environment are under great pressure. Given China is a traditional agricultural country, food production must also be accelerated to meet the needs of its growing population. Reducing agricultural water consumption and improving water use efficiency has become an urgent necessity to alleviate the water shortage in agricultural production.

China's water resources are unevenly distributed in time and space, and the supply and demand are unbalanced among regions. The country has huge agricultural water needs, low water efficiency, serious pollution problems, and pronounced regional differences. Numerous studies have analyzed the water use efficiency of the planting industry and proposed a number of suggestions, such as optimizing irrigation technology to maximize local crop yield^1–4^. In the quantification and evaluation of water resources efficiency, many methods have been adopted, including fuzzy comprehensive evaluation method^4,5^, analytic hierarchy process^6–15^, data envelopment analysis method^1,16–20,46^, and stochastic frontier method^3,21,22^. Researchers have proposed the variable fuzzy evaluation model based on information entropy, which combines the entropy weight method and fuzzy matter-element method to evaluate agricultural water use efficiency^4,5^. The model uses data envelopment analysis (DEA) to evaluate agricultural water resource efficiency and regional differences and establishes the Tobit regression model to analyze the influencing factors of agricultural water resource efficiency under different constraints^19,46^. The efficiency of agricultural water resources calculated by the stochastic frontier method often changes with the efficiency calculated by data envelopment analysis. Few people have used comparative studies to analyze how the inclusion or exclusion of unexpected output affects the calculation efficiency of agricultural water resources. DEA model does not need to set the form of the production function, which is more suitable for a multi-input and multi-output model with unexpected output. This method evaluates agricultural water use efficiency based on the influence of input–output variables. In selecting input–output variables, the study mainly uses resource endowment, economic level, and government expenditure to evaluate water resources utilization efficiency from the aspects of the natural environment, technical conditions, water resources consumption, and economic output^10,23–29^.

Most previous studies have mainly focused on high water consumption of agricultural industry, lack of objective evaluation basis for sustainable development of water resources and sustainable development of planting, forestry, animal husbandry, and fishery economy. In this study, we used agriculture, forestry, animal husbandry, and fishery data (hereinafter referred to as agriculture) in 31 provinces of China (except Hong Kong, Macao, and Taiwan). Given that the traditional DEA model is unable to distinguish multiple decision-making units, the super efficient SBM model is selected. The provincial differences in agricultural water use efficiency in China from 2007 to 2018 were analyzed quantitatively. The threshold model was established to determine which factors have a significant impact on agricultural water use efficiency, and the impact of these factors on resource efficiency under environmental constraints was assessed. Unlike previous water use efficiency calculations, the reference variables used in the study were chemical oxygen demand emissions, ammonia nitrogen emissions, and agricultural carbon emissions under environmental constraints. The results of this study can be used to improve the water use efficiency of planting, forestry, animal husbandry, and fishery, especially in high water demand areas. The conclusions and recommendations from this study can help improve water resource allocation, provide theoretical support and decision-making reference for agricultural production, and mitigate agricultural water pollution.

## Materials and research methods

### Study area

China is one of the countries with the poorest per capita water resources in the world while also having the largest water consumption in the world. In 2018, China's total water consumption was 601.55 billion m^3^, with 369.31 billion m^3^ of water used in agriculture, accounting for 61.4% of the total water^2^. Agriculture is the most important industrial sector in water resource consumption. However, due to regional and climate differences, the distribution of agricultural water resources is uneven, and the shortage of water resources seriously affects agricultural development in water-deficient areas.

**Figure 1:**
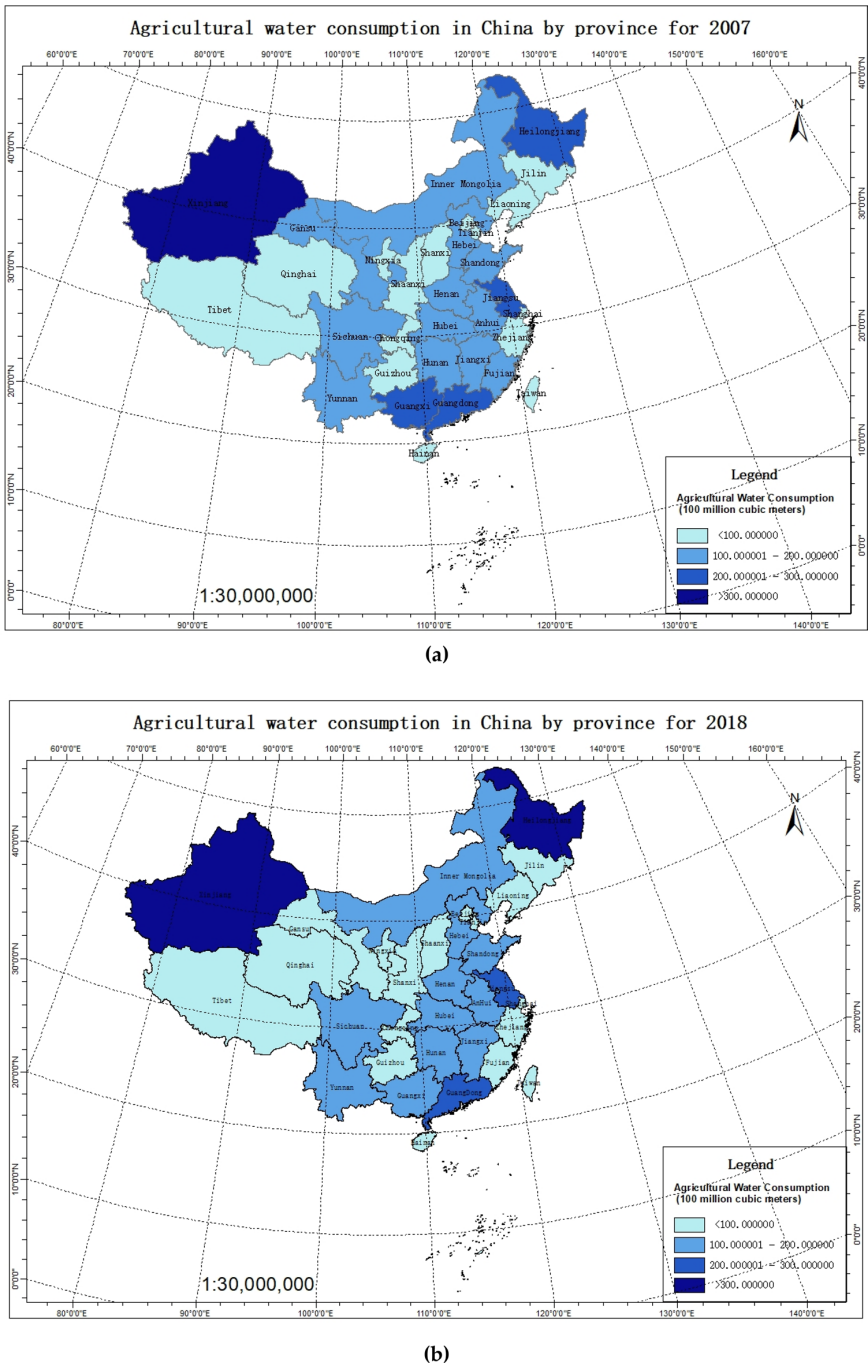
Agricultural water consumption in China by province for** (a) **2007 and** (b) **2018. Note: Map created using ArcGIS [10.2], (http://www.esri.com/software/arcgis).

Figure 1 shows the agricultural water consumption in China by province for 2007 and 2018. The agricultural water consumption includes farmland irrigation water consumption (classified as paddy field, irrigated land, vegetable field, groundwater exploitation), forest, animal husbandry, fishery, and livestock (classified as forest and fruit, grassland, fish pond, animal husbandry, groundwater exploitation), domestic water consumption of rural residents and rural ecological environment water consumption. Previous studies have mainly considered the irrigation water consumption of the planting industry as the research object at the provincial or regional levels (e.g., eastern, central, and western regions). Few were able to consider all 31 provinces in China and have comprehensively assessed water consumption and water use efficiency in the various types of agricultural production^3–6,10,16,17,22–25,30^. In this study, the agricultural water use efficiency and its influencing factors are assessed based on the agricultural water consumption of agriculture, forestry, animal husbandry, and fishery in China.

### Research method

In this study, the agricultural water use efficiency (under the common frontier and the group frontier) is calculated using the super-efficiency slacks-based measure (Super-SBM) model. The significant factors affecting water-use efficiency are then analyzed through the threshold regression model.

#### Super-efficiency SBM model

Data envelopment analysis (DEA) is an efficiency evaluation method proposed by Charnes^31^, a famous American operational research scientist. While traditional radial and angular DEA models do not require the specific form of the estimation function, they ignore the relaxation of variables and result in efficiency values in the range of 0 to 1. If there are multiple efficiency value of decision making units(DMUs) with an efficiency value of 1, these values cannot be compared. The efficiency of the super efficiency DEA model can be greater than 1, which means that the efficiency level of all decision-making units can be compared.

To avoid the problem of slack variables, Tone (2001) proposed the SBM model, which is a non-radial and non-angular DEA analysis method based on the relaxation variable measure^16–20,32^. The SBM model of unexpected output solves the slack problem of input and output variables, minimizing deviations in the efficiency measurement. The super-efficiency SBM model combines the super-efficiency DEA model and the SBM model. It is also one of the methods based on data envelopment analysis, which can measure the efficiency of all decision-making units and the slack of input and output variables.

Assume n to be the decision-making units, each of which has m inputs, expected output r_1_, and unexpected output r_2_. Let X (X ∈ R^m^), Y^d^ (Y^d^ ∈ R^s1^), and Y^u^ (Y^u^ ∈ R^s2^) be matrices, such that $X=[{x}_{1},\dots ,{x}_{n}]\in {R}^{m*n}$ and $Y=[{y}_{1}^{d}, \dots ,{ y}_{n}^{d}\in {R}^{{r}_{1}*n}$. The form of the super-efficiency SBM model is as follows^1,17,19,54^:

1 $$min=\frac{\frac{1}{m}\sum_{i=1}^{m}(\overline{x}/{x}_{ik})}{1/\left({r}_{1}+{r}_{2}\right)*(\sum_{r=1}^{{r}_{1}}\overline{{y}^{d}}/{y}_{rk}^{d}+\sum_{q=1}^{{r}_{2}}\overline{{y}^{u}}/{y}_{qk}^{u}}.$$

Among them,

2 $$\overline{x}\ge \sum_{j=1\ne k}^{n}{x}_{ij}{\lambda }_{j}, i=1,\dots ,m;$$

3 $$\overline{{y}^{d}}\le \sum_{j=1,\ne k}^{n}{y}_{rj}^{d}{\lambda }_{j}, r=1,\dots ,{s}_{1};$$

4 $$\overline{{y}^{d}}\ge \sum_{j=1,\ne k}^{n}{y}_{qj}^{u}{\lambda }_{j}, q=1,\dots ,{s}_{2};$$

5 $${\lambda }_{y}\ge 0,j=1,\dots ,n;j\ne 0;$$

6 $$\overline{x}\ge {x}_{k},k=1,\dots ,m;$$

7 $$\overline{{y}^{d}}\le {y}_{k}^{d},d=1,\dots ,{r}_{1};$$

8 $$\overline{{y}^{u}}\ge {y}_{k}^{u},b=1,\dots ,{r}_{2}.$$

Based on the Super-SBM model (Eq. 1) and its constraint formula, the agricultural water use efficiency for the different provinces was calculated for the period 2007–2018 using Maxdea 8 ultra software.

#### Threshold effect

Considering the differences in economic development and technical levels, the agricultural water use in different regions of China shows characteristics of time-series evolution, spatial heterogeneity, and unbalanced spatial distribution. There is a non-linear relationship between the influencing factors of agricultural water use efficiency, which suggests the existence of certain threshold characteristics^33,34^. This means that for a particular determinant, agricultural water use efficiency would be affected differently depending on whether the parameter has crossed the threshold. In this study, the threshold panel model proposed by Hansen is used. The threshold value of the threshold variable is taken as the critical point, and the regression equation is divided into different stage intervals to analyze the influence of threshold variables on the explained variables at different stages . Therefore, according to the relationship between agricultural water use efficiency and its influencing factors in different regions, the following single threshold regression model is set:

9 $${Y}_{it}=\alpha {X}_{it}+{\beta }_{1}{T}_{it}I\left({T}_{it}\le {\gamma }_{1}\right)+{\beta }_{2}{T}_{it}I\left({T}_{it}>{\gamma }_{1}\right)+C+{\varepsilon }_{it},$$

such that i is the province; t is the year; Y_it_ and T_it_ are the explanatory variables and explained variables, respectively; X_it_ is the control variable that has a significant impact on the explained variables; Tit is threshold variable, which changes with the different explanatory variables; γ is a specific threshold value; α is the corresponding coefficient vector; β_1_ and β_2_ represent the influence coefficients of the threshold variable T_it_ on the explained variable Y_it_ in the case of ${T}_{it}\le {\gamma }_{1}$ and ${T}_{it}>{\gamma }_{1}$ , respectively; C is a constant; ε is random disturbance term, ${\varepsilon }_{it}\sim i.i.d.N(0,{\sigma }^{2})$; and, I (·) is an indicative function. After obtaining the estimated value of each parameter, two tests need to be carried out: (1) establish whether the threshold effect is significant; and (2) determine whether the estimated threshold value is equal to the true value. In addition, the above equation assumes that only one threshold exists. For two or more thresholds, the model would have to be adjusted according to the data.

Based on the panel data of 31 provinces in China from 2007 to 2018^44–46^, Stata15.0 software was used to perform threshold regression on seven variables: per capita water resources, rural labor force, disposable income, government's attention, foreign trade dependence, industrial structure, and gross domestic product (GDP). The threshold effect of each factor can be analyzed, and the impact on agricultural water consumption can be assessed using the threshold value.

### Variable selection and data source

The super-efficiency SBM model was used in calculating the agricultural water use efficiency for the 31 provinces in China from 2007 to 2018. The input–output indicators were defined before the calculations, as shown in Extended Data Table 1.

The selection of input–output factors to measure the utilization efficiency of agricultural water resources follows the principles of availability and operability. The input variables included: (1) agricultural water consumption, (2) the number of employees in agriculture, forestry, animal husbandry, and fishery, (3) the total power of agricultural machinery, and (4) the expenditure of local finance on agriculture, forestry, and water affairs. In terms of output, the added value in agriculture, forestry, animal husbandry, and fishery (based on 2007) was used as the expected output, while ammonia nitrogen emission, agricultural chemical oxygen demand emission, and agricultural carbon emission comprised the unexpected output.

This study considered the scale of carbon emissions released by the agricultural system. According to existing research, agricultural carbon emissions are associated with rural environmental pollution^35^. The main consequence of agricultural pollutant emissions is soil pollution, which leads to rural groundwater pollution^36^,^37–41^. The deterioration of groundwater quality adversely affects the development of the agricultural economy and threatens the safety of the drinking water supply for rural residents.

The threshold regression model was used to investigate the convergence of agricultural water use efficiency and observe the changes in agricultural water consumption under different influencing factors. The control variables include the following: water resource endowment, the number of agricultural labor, the income level of rural residents, industrial structure, the degree of government's attention, the degree of dependence on foreign trade, and the level of economic development, as shown in Extended Data Table 2. For water resource endowment (WR), WR is expressed in per capita water resource (m^3^ / person). Zhang Lixiao^45,46^ and previous studies have shown a negative correlation between water resource endowment and water resource utilization. For agricultural labor (ah), the variable is expressed by the number of people engaged in agriculture, forestry, animal husbandry, and fishery (10,000 people). Past studies suggest rural population affects the consumption of agricultural water resources^47–52^. For income levels, rural residents' income level is indicated by the per capita disposable income of rural households. Wang Xueyuan et al.^3^ and Han Qing et al.^53^ argue that the increase in the rural residents' income would limit agricultural water consumption. For industrial structure (× 2), which is expressed by the proportion of industrial added value in GDP, research has shown water resource efficiency would vary under different industrial structures^54–56^. For the government's attention degree (GA), the variable is expressed by the proportion of agriculture, water affairs, and forestry spending in the total financial expenditure. The government's support for comprehensive agricultural development and infrastructure and technology upgrading for agricultural, forestry, and water conservation significantly affects water resource utilization efficiency^16,56–58^. For the degree of dependence on foreign trade (open), the parameter is indicated by the proportion of the total import and export of agricultural and sideline products in the GDP. Changes in import demand can reduce or increase the consumption and pollution of water resources. Likewise, export demand changes, especially in high water-consuming and high polluting products, can significantly improve or degrade water resource efficiency. And for the level of economic development, expressed in terms of GDP, the level of regional economic development plays a positive role in promoting the efficiency of water resource utilization^59–61^.

## Empirical results and analysis

### Descriptive statistics of each variable

Based on the data available, six indicators were selected to describe agricultural water use efficiency. The average value, standard deviation, minimum value, and maximum value of each variable for each of China's 31 provinces were calculated and analyzed. As shown in Extended Data Table 3, there is a large gap in agricultural water use indicators among provinces. This suggests that all indicators have significantly changed in the past 12 years and highlights the need to analyze the change characteristics of agricultural water use efficiency in various provinces.

### Analysis of super-efficiency SBM model

**Figure 2:**
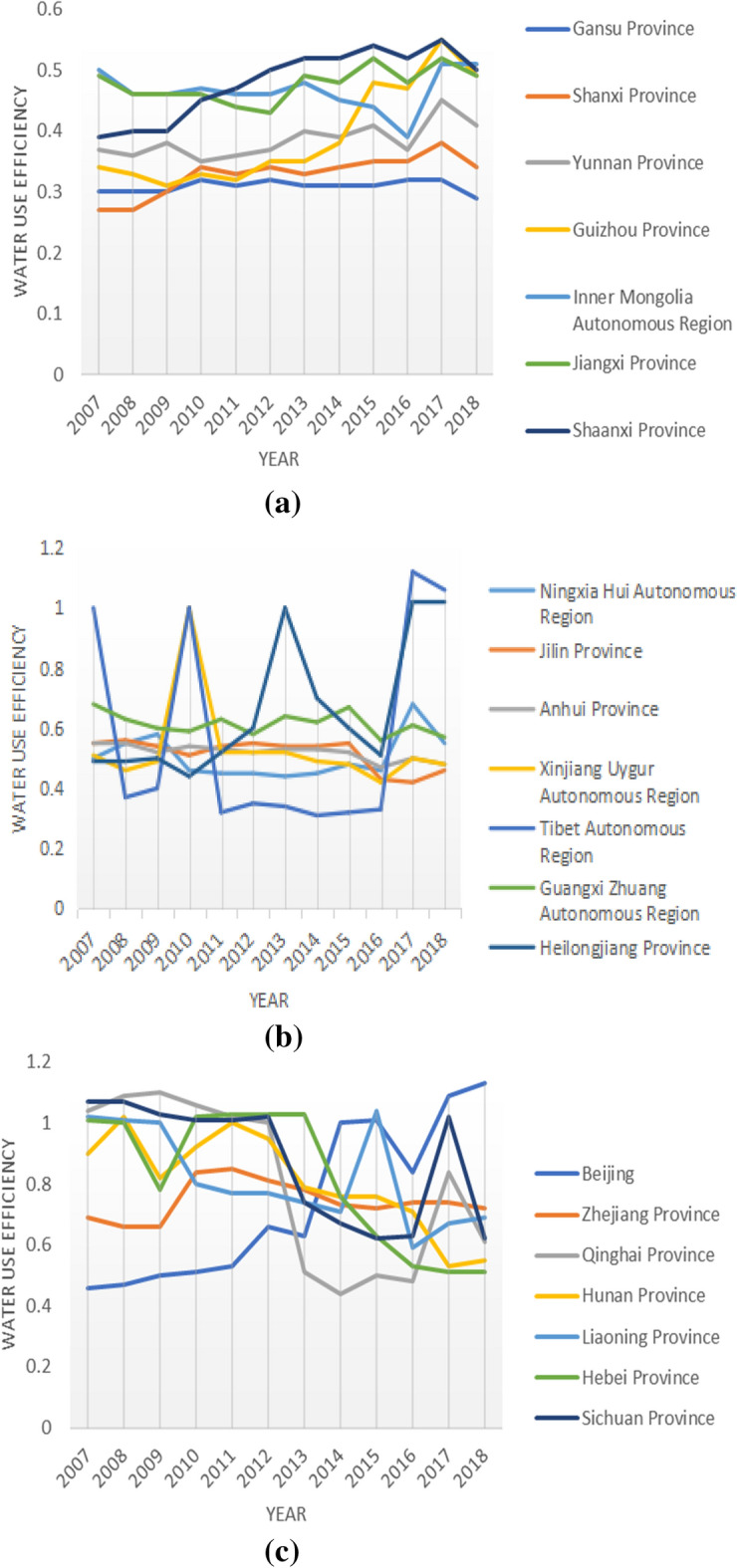

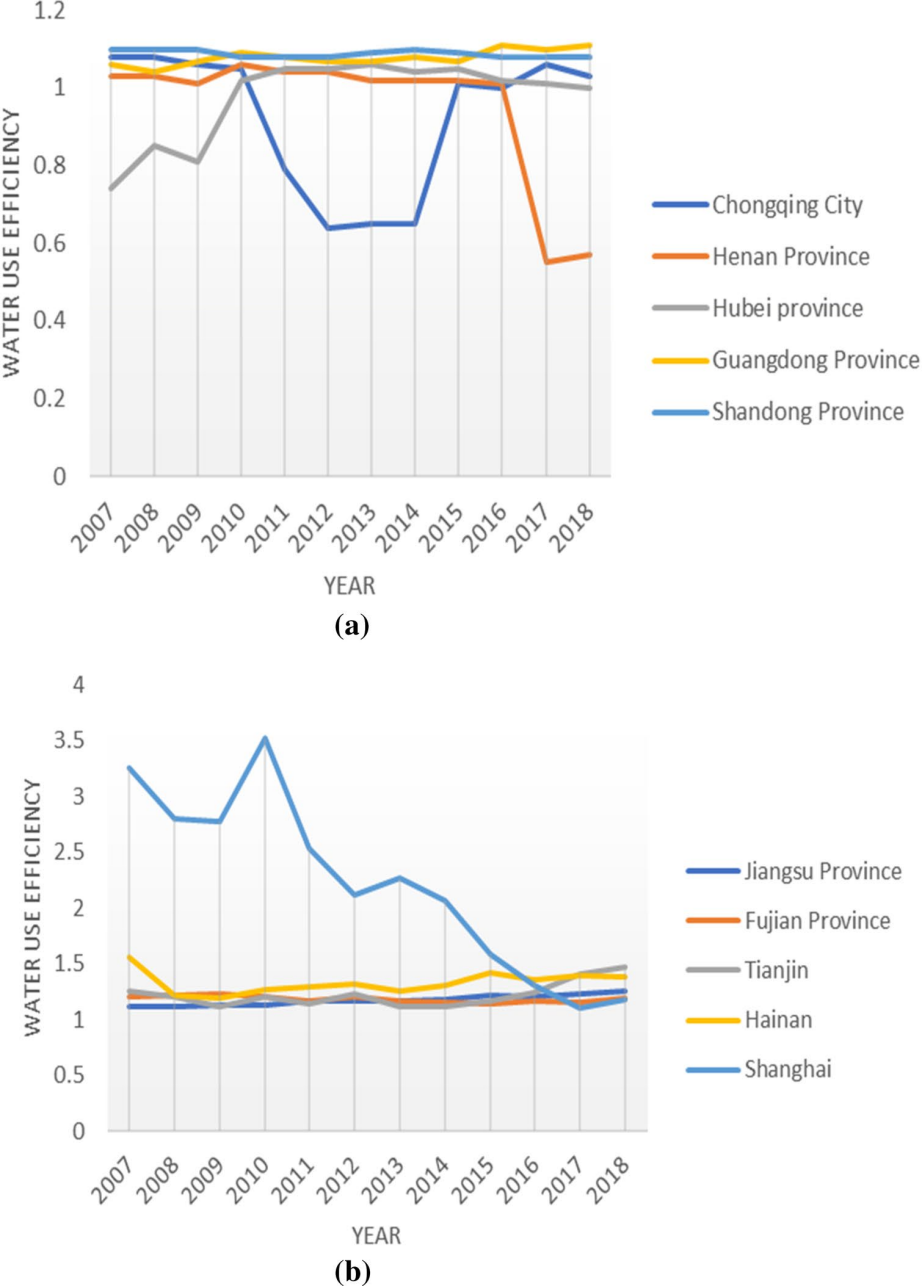
Evaluation of agricultural water use efficiency in different regions. This figure shows the agricultural water use efficiency of 31 provinces, autonomous regions, and cities in China from 2007 to 2018. The above five charts are listed separately according to the average water use efficiency of **(a)** 0.3–0.5, **(b)** 0.5–0.7, **(c)** 0.7–0.9, **(d)** 0.9–1.1, and **(e)** 1.1 and above.

The results are shown in Fig. 2. At the national level, the national average of agricultural water resource efficiency from 2007 to 2018 is non-DEA efficient, and that over time, the national average shows a fluctuating but generally decreasing trend. Sixteen provinces also had a fluctuating downward trajectory in agricultural water use efficiency, namely Hebei, Liaoning, Jilin, Shanghai, Anhui, Fujian, Shandong, Henan, Hunan, Guangxi, Hainan, Chongqing, Sichuan, Gansu, Qinghai, and Xinjiang. Shanghai exhibited a relatively large change in agricultural water efficiency, having an efficiency difference of 2.07 in 11 years. This is mainly due to Shanghai's prioritization towards industrialization, as highlighted by the annual decrease in the city's employment in agriculture, forestry, animal husbandry, and fishery. The annual agricultural water consumption in Shanghai only accounts for 15% of the region's total water consumption. The agricultural water use efficiency showed an upward trend in 15 regions and is most significant in Beijing and Heilongjiang. In Heilongjiang Province, the agricultural water consumption accounts for 88% of the total water consumption, and the added value of the primary industry accounts for a large proportion of the GDP. This is because, in Heilongjiang, the government pays considerable attention to improving agricultural water use efficiency. In Beijing, improvements in agricultural water use efficiency are related to the intensification of rural reforms implemented in recent years.

In terms of the spatial dimension, the agricultural water use efficiency in coastal areas was significantly higher than in other regions, highlighting the agricultural, economic, and technological progress of Fujian, Guangdong, and Hainan. In arid and semi-arid areas, the efficiency levels are generally low, resulting in relatively small differences within these provinces. In areas with less precipitations with comparatively backwards agricultural water technology and low management experiences (e.g., Gansu, Shanxi, Jilin, Xinjiang, and Anhui), their water resource efficiency levels are low. In provinces with relatively more water resources, such as Hubei, Anhui, Jiangxi, and Hunan, the backward agricultural irrigation technology and management system have resulted in water resource efficiency levels much lower than the national average. In Chongqing, Guangxi, Sichuan, Guizhou, and Yunnan, rainfall is sufficient but their utilization rates of water resources are low^8^. Thus, while their efficiency levels are higher than in other regions, the absolute differences are not as pronounced due to the low overall level.

### Threshold effect

The results of the threshold effect test are summarized in Extended Data Table 4. The water resource per capita, disposable income, foreign trade dependence, industrial structure, and gross domestic product (GDP) pass the threshold test under the significant level of 10% for agricultural water consumption, and the optimal threshold value is 1, which indicates that these five parameters have a single threshold effect. On the other hand, the number of rural labor force and the degree of government attention did not pass the parameter test, indicating that there is no threshold effect phenomenon.

The regression results of the threshold panel model at the national level (Extended Data Table 4) show that the decrease in per capita water resources, disposable income, foreign trade dependence, industrial structure, and gross domestic product (GDP) is conducive to restraining the growth of agricultural water consumption.

In terms of per capita water resources, when the per capita value is lower than the threshold value of 3892.69M^2^ per person, the agricultural water use efficiency and agricultural water consumption change in the same direction. As a big agricultural country, China has been facing water shortage problems for a long time, and water users have relatively strong awareness of the importance of water conservation. Thus, agricultural water users become more likely to transition from high water-consuming products to those that require less water use. This directly and effectively restrains the consumption of water resources. From the perspective of per capita disposable income, when the value is lower than the threshold value of 21,125.00 yuan, reducing agricultural water use efficiency also inhibits agricultural water consumption. In China, the use of agricultural water entails specific payment (water fees), the price of which is set by the government. Changes in the per capita disposable income can directly or indirectly affect water consumption. In general, agricultural water consumption is inversely related to agricultural water prices, such that an increase in water prices would result in decreased water consumption. In terms of foreign trade dependence, when the ratio of total import and export to GDP is less than 0.31%, reducing the import and export trade of agricultural and sideline products would also reduce agricultural water consumption. This is in line with China's export of land- and labor-intensive agricultural products, which, as a major agricultural country, directly promotes the consumption of water resources. According to the data in foreign trade dependence for each province, the ratio of total import and export to GDP has been rising in recent years, which is closely related to China's increasing competitiveness in foreign trade. In terms of industrial structure and GDP, with the steady development of the social economy, China has been vigorously advocating for the development of the tertiary industry, while its primary and secondary industries implement industrial transfer and upgrading. At the same time, as the provinces become heavily economically reliant on secondary industries, they become less dependent on primary industries, thus decreasing water consumption from agriculture, forestry, animal husbandry, and fishery.

## Discussion

### Environmental constraints affecting agricultural water use efficiency

China is currently dealing with a weak agricultural foundation, exacerbated by intensified resource and environmental constraints. Resource waste, soil and water pollution, ecological damage, and other problems seriously restrict sustainable development in agriculture. Through empirical research and analysis, this study found that agricultural water resource utilization efficiency had a general downward trend. This result is different from the findings of past studies^4,16–19,37,45,48,54^ because this study focuses on the agriculture, forestry, animal husbandry, and fishery industries in China. In calculating water resource utilization efficiency, traditional agricultural water pollution parameters (chemical oxygen demand emissions, ammonia nitrogen emissions) and agricultural carbon emissions were added as the unexpected output variables.

In terms of water pollution, China has implemented agricultural and rural domestic sewage treatment. However, detailed and systematic statistical data on rural environmental pollution control investment and agricultural water environment quality are still being overlooked. Thus, due to limited data availability, only a few environmental pollutants were included in this study, which should be expanded and improved in future research. Strengthening China's agricultural infrastructure, imposing much-needed environmental regulations, and developing strategies that would break resource and environmental constraints are required to comprehensively improve the efficiency of agricultural production and enhance the agricultural industry's institutional development.

### Foreign trade dependence on agricultural water use

To further understand the impact of particular factors on agricultural water use efficiency, various parameters were evaluated: per capita water resources, disposable income, foreign trade dependence, primary industry structure, and GDP. In contrast with previous studies, agricultural foreign trade dependence was examined as a potential determinant for agricultural water use. The findings show that reducing the dependence on foreign trade of agricultural and sideline products would results in decreased agricultural water consumption. And based on the data on foreign trade dependence of various provinces, the ratio of total import and export to GDP is on the rise.

As a traditional agricultural country, the import and export trade of agricultural products is a critical component of the national economy. Due to China's pivot towards greater agricultural trade liberalization, transnational trade has significantly increased, rapid export growth in labor-intensive products (such as vegetables, fruits, livestock products) has been sustained, and import growth in land-intensive agricultural products (such as grain, cotton, and oil) has accelerated. These agricultural products directly consume water resources, from planting and breeding to processing and marketing. Considerable attention should be given to better understand the relations and interdependence of foreign trade and agricultural production industries, which could be considered in further research.

## Conclusion and recommendations

This study evaluated the water resource utilization efficiency of agriculture, forestry, animal husbandry, and fishery in China and quantitatively analyzed agricultural water resource consumption and water environment degradation. Using super efficiency SBM analysis, the main agricultural pollution emissions (COD emissions, ammonia nitrogen emissions, agricultural carbon emissions) were proposed as environmental constraints for the first time. By constructing seven different explanatory variables, the threshold regression model was used to measure the impact of agricultural water use efficiency on agricultural water consumption. As far as we know, few people have implemented comparative studies to analyze how the inclusion or exclusion of unexpected output affects the efficiency level evaluation of agricultural water resources, particularly environmental factors, such as cod, TN, TP, and C emissions.

The results of agricultural water resource efficiency evaluation provide some useful enlightenment. Coastal areas (Fujian, Guangdong, Hainan) are economically and technologically developed areas. Due to the high level of labor and the wide range of agricultural machinery promotion, agricultural water resource utilization efficiency is generally higher than in other areas. Due to the continued changes in industrial structure, the agricultural water use efficiencies in Beijing, Heilongjiang, Hubei, and Tianjin have also been rising. In contrast, in arid and semi-arid areas with less precipitation (e.g., Gansu, Shanxi, Jilin, Xinjiang, and Anhui), agricultural water use efficiency is generally low mainly because their agricultural water use technology and management experience are less developed.

Through the threshold model, we found that the single threshold's influence coefficient is positive and significant. This means that when the per capita water resource value is higher than the threshold of 3892.69 M^2^, the per capita disposable income is higher than the threshold of 21,125.00 yuan. If the total import and export ratio to GDP is less than 0.31%, agricultural water use efficiency is improved, and agricultural water use declines. If not, the effect is not pronounced and can result in adverse effects.

We recommend implementing water pricing mechanism reforms to promote rural drinking water safety, enhance awareness in water-saving measures, and adopt policies protecting the water supply structure and improving the irrigation system. Especially in technologically backward areas, subsidies for grain production mechanization and agricultural equipment transformation should be expanded and promoted. The discharge standards of water pollutants for agriculture, forestry, animal husbandry, and fishery should be regularly reviewed and reformed to cultivate new driving forces for development. Policy reforms and measures should be put in place, focusing on developing new technologies and new models that would accelerate the improvement of air and water quality standards and support the long-term economic and environmental goals of the region.

Due to the limitations of existing data, further research is needed. Although this study proposed modifications and improvements to the agricultural water use efficiency system, more improvements are needed. For instance, current research has often overlooked national and regional differences, which could be analyzed in future studies.

## Supplementary Information

Supplementary Tables.

## References

[1] Hong, M. & Zheng, L. regional differences and spatiotemporal characteristics of agro ecological efficiency in China. *Stat. Decis.* **36**, 56–61 (2020).

[2] Duan, A. & Zhang, J. Study on water use efficiency of grain crops in irrigated farmland in China. *Acta agri engi neering Sinica* **16**, 41–44 (2000).

[3] Wang, X. & Zhao, L. Agricultural water use efficiency and its influencing factors in China: SFA analysis based on provincial panel data from 1997 to 2006. *Issues Agric. Econ.* **29**, 10–18 (2008).

[4] Jiao, Y. & Zhu, M. Estimation of agricultural water use efficiency based on variable fuzzy evaluation of information entropy. *Water Saving Irrig.* **221**, 80–83 (2014).

[5] Yang, D., Tang, Y. & Tang, D. Application of fuzzy matter element method based on entropy weight in agricultural water use efficiency evaluation. *Water Saving Irrig.* **278**, 64–67 (2018).

[6] Cao X, Zeng W, Wu M, Guo X, Wang W (2020). Hybrid analytical framework for regional agricultural water resource utilization and efficiency evaluation. Agric. Water Manag..

[7] Ghosh, D., Mandal, M., Banerjee, M. & Karmakar, M. Impact of hydro-geological environment on availability of groundwater using analytical hierarchy process (AHP) and geospatial techniques: A study from the upper Kangsabati river basin. *Groundwater Sustain. Dev.* **11**, 100419 (2020).

[8] Li G, Cheng Y, Yan S, Mou H, Meng Q (2019). Study on comprehensive evaluation of water conservancy modernization degree based on AHP and fuzzy comprehensive evaluation method. Res. Water Conserv. Dev..

[9] Shang, M. Evaluation of influencing factors of rural drinking water safety based on analytic hierarchy process. *Water Conserv. Technol. Econ*. **25**, 39–42 (2019).

[10] Feng Z, Liu B, Yang Y (2005). Trend analysis and data reconstruction of cultivated land resources in China: 1949–2003. J. Nat. Resour..

[11] Charnes, A., Cooper, W. W. & Rhodes, E. Measuring the efficiency of decision making units. *Eur. J. Oper. Res.* **2**, 429–444 (1978).

[12] Azizi, H. & Ajirlu, H. G. Measurement of the worst practice of decision-making units in the presence of non-discretionary factors and imprecise data. *Appl. Math. Model.* **35** 4149–4156 (2011).

[13] Wang, Y. B., Wu, P. T., Engel, B. A. & Sun, S. K. Application of water footprint combined with a unified virtual crop pattern to evaluate crop water productivity in grain production in China. *Sci. Total Environ.* **497**, 1–9 (2014). 10.1016/j.scitotenv.2014.07.089 25112819

[14] Hu, D. & Zhan, S. Study on the construction of comprehensive evaluation system of water saving society construction. *Jiangsu Water Res.* **268**, 24–29 (2019).

[15] Saaty, T. L. The modern science of multicriteria decision making and its practical applications: The AHP/ANP approach. *Oper. Res.* **61**, 1101–1118 (2013).

[16] Wang G, Lin N, Zhou X, Li Z, Deng X (2018). Three-stage data envelopment analysis of agricultural water use efficiency: A case study of the Heihe River Basin. Sustainability.

[17] Zhao, M. & Liu, S. Evaluation of agricultural water use efficiency based on double frontier sbm-dea model. *Water Econ*. **38**, 58–64+71+91 (2020).

[18] Chen X, Zhang Y, Liu L, Wang L (2020). Evaluation of water resources utilization efficiency in Shaanxi and Shanxi Province based on super efficiency DEA model. J. Irrig. Drainage.

[19] Yu L, Yang S (2019). Environmental efficiency evaluation of agricultural water resources in China based on super efficiency SBM and analysis of its influencing factors. Rural Water Conserv. Hydropower China.

[20] Tone K (2001). A slacks-based measure of efficiency in data envelopment analysis. Eur. J. Oper. Res..

[21] Song, M., Li, M. & Zhang, F. Impact of irrigation and water conservancy investment on agricultural total factor productivity based on spatial effect. *Water Econ*. **38**, 46–52+90–91 (2020).

[22] Laajimi A, Guesmi A, Mahfoudhi A, Dhehibi B (2009). Analyzing supply response of fruit tree products in Tunisia: The case of peaches. Agric. Econ. Rev..

[23] Yanling Hu, Zhiguo C, Zhenguo L (2015). Study on agricultural water use efficiency in Hebei Province based on entropy method. Agric. Resour. Regional. China.

[24] Fan, Y., Wang, C. & Nan, Z. Comparative evaluation of crop water use efficiency, economic analysis and net household profit simulation in arid Northwest China *Agric. Water Manag.* **146**, 335–345 (2014).

[25] Kim, S. H., Choi, S. H., Koo, J. Y., Choi, S. I. & Hyun, I. H. Trend analysis of domestic water consumption depending upon social, cultural, economic parameters. *Water Supply* **7**, 61–68 (2007).

[26] Azad, M. A. S. & Ancev, T. Measuring environmental efficiency of agricultural water use: A Luenberger environmental indicator. *J. Environ. Manag.* **145**, 314–320 (2014). 10.1016/j.jenvman.2014.05.037 25103337

[27] Xiong, Y., Peng, S., Luo, Y., Xu, J. & Yang, S. A paddy eco-ditch and wetland system to reduce non-point source pollution from rice-based production system while maintaining water use efficiency. *Environ. Sci. Pollut. Res.* **22**, 4406–4417 (2015). 10.1007/s11356-014-3697-7 25304242

[28] Li, X., Jiang, W., Yang, Y. & Feng, X. Spatial heterogeneity and main controlling factors of water conservancy green development in China. *South North Water Divers. Water Conserv. Sci. Technol.* **18**, 191–200 (2020).

[29] Shang J, Wei D, Ji X (2020). Technological progress, agricultural water use efficiency and rebound effect—An empirical study based on panel data of major grain producing areas in China. Ecol. Econ..

[30] Jiang W, Liu J, Hu H (2020). Study on temporal and spatial evolution of forestry ecological efficiency and threshold effect of environmental regulation in China. J. Central South Univ. For. Sci. Technol..

[31] Huang, C. Analysis on the utilization efficiency and influencing factors of agricultural water resources in Xinjiang, Shihezi University (2019).

[32] Tone K (2002). A slacks-based measure of super-efficiency in data envelopment analysis. Eur. J. Oper. Res..

[33] Hansen BE (1999). Threshold effects in non-dynamic panels: Estimation, testing, and inference. J. Econ..

[34] Van, B. D., Sameh, K. & Tetsuya, S. Changes to long-term discharge and sediment loads in the Vietnamese Mekong Delta caused by upstream dams. *Geomorphology* **353** (2020).

[35] Li, J., Li, H. & Xie, L. Potential, efficiency and influencing factors of agricultural pollution reduction in China. *Agric. Technol. Econ.* 118–126 (2012).

[36] Cheng, S. China's Green Industrial Revolution: An explanation from the perspective of environmental total factor productivity (1980–2008). *Econ. Res*. **45**, 21–34+58 (2010).

[37] Azad AS, Ancev T (2014). Measuring environmental efficiency of agricultural water use: A Luenberger environmental indicator. J. Environ. Manag..

[38] Shi, Z., Huang, H., Wu, Y., Chiu, Y.-H. & Qin, S. Climate change impacts on agricultural production and crop disaster area in China. *Int. J. Environ. Res. Public Health*10.3390/ijerph17134792 (2020). 10.3390/ijerph17134792 PMC7369862 32635256

[39] Yue, l. & Wang, X. Analysis of agricultural technical efficiency and total factor productivity in China from the perspective of environmental regulation—Based on distance function.* Jilin Univ. J. Soc. Sci. Edn*. **53**, 85–92 (2013).

[40] Ma H, Ding Y, Wang L (2017). Measurement and convergence analysis of green water resources utilization efficiency. J. Nat. Resour..

[41] Sun, C., Chen, L. & Liu, Y. Estimation of crop green water occupation index and analysis of its spatial and temporal differences in China. *Prog. Water Sci.* **21**, 637–643 (2010).

[42] NBS.National Data. *National Bureau of Statistics*. https://data.stats.gov.cn/easyquery.htm?cn=A01 (2019).

[43] Shan Y, Guan D, Zheng H (2018). China CO_2_ emission accounts 1997–2015. Sci. Data.

[44] Shan Y, Huang Q, Guan D (2020). China CO_2_ emission accounts 2016–2017. Sci. Data.

[45] Liu, Y. & Wang, J. Analysis of agricultural water resources utilization efficiency—Application of target ratio of total factor water resources adjustment. *J. Huazhong Agric. Univ. (social science edition)* **152**, 26–30 (2012).

[46] Liu, Y., Wang, K. L., Meng, X. R. & Yang, B. C. Spatial and temporal disparities and dynamic evolution of industrial water resources efficiency in the Yangtze river economic belt. *Water Resour. Power* **36**, 55–59 (2018).

[47] Xia, l., Shi, X., Feng, S. & Qu, F. Analysis on the influencing factors of farmers' water resources utilization efficiency under the background of agricultural industrialization—Based on the empirical analysis of Minle County in Gansu Province. *China's Popul. Resour. Environ.* **23**, 111–118 (2013).

[48] Pan J (2016). Regional differences and influencing factors of agricultural water use efficiency in China. Hubei Agric. Sci..

[49] Tian, F., Zhang, Y. & Lu, S. Spatial-temporal dynamics of cropland ecosystem water-use efficiency and the responses to agricultural water management in the Shiyang River Basin, northwestern China. *Agricul. Water Manag.* **237**, 106176 (2020).

[50] Tuqan, N., Haie, N. & Ahmad, M. T. Assessment of the agricultural water use in Jericho governorate using sefficiency. *Sustainability*10.3390/su12093634 (2020).

[51] Yang, Z. & Wei, X. Analysis of the total factor energy efficiency and its influencing factors of the belt and road key regions in China. *Environ. Sci. Pollut. Res.* **26**, 4764–4776 (2019). 10.1007/s11356-018-3961-3 30565113

[52] Xu L, Huang Y (2012). Analysis of agricultural irrigation water use efficiency and its influencing factors—Based on field investigation in Mengcheng County, Anhui Province. Resour. Sci..

[53] Qing H (2011). yuan XueGuo, impact of participatory irrigation management on Farmers’ water use behavior. Popul. Resour. Environ. China.

[54] Ding X, He J, Wang L (2018). Study on provincial water resources utilization efficiency and driving factors considering undesirable output—Based on se-sbm and Tobit model. China's Popul. Resour. Environ..

[55] Wanowaa, Y., Furaha, R. K., Yan, L. & Wei, D. Effects of planting field on groundwater and surface water pollution in China. *Clean Soil Air Water* **48**, 1900452 (2020).

[56] Qasemipour, E. & Abbasi, A. Assessment of agricultural water resources sustainability in arid regions using virtual water concept: Case of South Khorasan Province, Iran. *Water*10.3390/w11030449 (2019).

[57] Li, J. & Ma, x. Water use efficiency and influencing factors of grain production in grain producing areas under the constraints of resources and environment. *Res. Agric. Modern.* **36**, 252–256 (2015).

[58] Francesco G, Meri R, Davide V (2013). Pricing policies in managing water resources in agriculture: An application of contract theory to unmetered water. Water.

[59] Yi, X., Wang, X. & Huang, J. Evaluation of coordination degree between regional water conservancy and social economy and analysis of spatial difference of comprehensive level. *Water Saving Irrig.* **212**, 57–63 (2013).

[60] Mei, L. & Chen, Z. The Convergence analysis of regional growth differences in China: The perspective of the quality of economic growth. *J. Serv. Sci. Manag.* **9**, 453–476 (2016).

[61] Gómez-Limón, J. A., Gutiérrez-Martín, C. & Montilla-López, N. M. Agricultural water allocation under cyclical scarcity: The role of priority water rights. *Water*10.3390/w12061835 (2020).

[62] Han, S. Technical approaches for efficient utilization of precipitation resources in Dryland farmland on the Loess Plateau. *Agric. Res. Arid Area* **20**, 1–9 (2002).

[63] Xue, J. *et al*. Assessing sustainability of agricultural water saving in an arid area with shallow groundwater. *Irrigation Drainage* **68**, 205–217 (2019).

