# Supplementary material for: A study on the effects of regional differences on agricultural water resource utilization efficiency using super-efficiency SBM model
Source: Sci Rep. 2021 May 11;11:9953. doi: 10.1038/s41598-021-89293-2 (PMC8113275; doi:10.1038/s41598-021-89293-2)
Supplement: Supplementary file 1 — Supplementary Tables. [file 41598_2021_89293_MOESM1_ESM.docx]

**Extended Data Table 1. Input-output index selection and selection basis**

Note: The carbon emission data of agriculture, forestry, animal husbandry, and fishery are from the Ceads database, and the rest are from the China Statistical Yearbook [46-49].

**Extended Data Table 2. Selection of control variables and data sources**

Note: The above data sources are all from China Statistical Yearbook; only awe's data source is calculated by super efficiency SBM, refer to table 2 .

**Extended Data Table 3. Descriptive statistical characteristics of data**

| **Threshold variable** | **Threshold effect** | **F value** | **Prob.** | **critical value** | | | **Threshold** | **95% confidence interval** |
| --- | --- | --- | --- | --- | --- | --- | --- | --- |
|  |  |  |  | **10%** | **5%** | **1%** |  |  |
| Per capita water resources | Single threshold | 79.38 | 0.03 | 42.29 | 63.18 | 85.26 | 3892.69 |  |
|  | Double threshold | 103.22 | 0.12 | 114.18 | 163.06 | 252.11 | 3892.69 |  |
|  |  |  |  |  |  |  | 1488.60 | [1484.66,1632.25] |
| Number of rural labor force | Single threshold | 35.00 | 0.15 | 39.63 | 45.97 | 55.55 | 1827.40 | [1810.15,1863.76] |
|  | Double threshold | 23.63 | 0.26 | 35.65 | 43.06 | 71.45 | 1827.40 | [1810.15,1856.60] |
|  |  |  |  |  |  |  | 876.27 | [858.93,881.88] |
| Disposable income | Single threshold | 38.59 | 0.04 | 28.13 | 35.22 | 52.12 | 21125.00 | [20706.90,21191.64] |
|  | Double threshold | 13.56 | 0.37 | 33.10 | 40.96 | 64.80 | 21125.00 | [20706.90,21191.64] |
|  |  |  |  |  |  |  | 5624.00 | [5082.70,5719.40] |
| Government attention | Single threshold | 24.11 | 0.12 | 25.12 | 28.52 | 40.69 | 6.26 | [6.21,6.33] |
|  | Double threshold | 11.57 | 0.47 | 25.71 | 33.01 | 53.68 | 6.26 | [6.21,6.33] |
|  |  |  |  |  |  |  | 14.52 | [14.09,14.60] |
| Foreign trade dependence | Single threshold | 44.33 | 0.02 | 27.66 | 32.18 | 47.10 | 0.31 | [0.30,0.31] |
|  | Double threshold | 23.11 | 0.14 | 25.44 | 28.43 | 45.53 | 0.31 | [0.30,0.31] |
|  |  |  |  |  |  |  | 1.50 | [1.42,1.50] |
| Industrial structure | Single threshold | 44.22 | 0.03 | 37.22 | 42.15 | 49.37 | 0.61 | [0.48,0.65] |
|  | Double threshold | 23.19 | 0.30 | 59.48 | 80.83 | 127.94 | 0.51 | [0.41,0.53] |
|  |  |  |  |  |  |  | 12.81 | [12.55,13.02] |
| Gross domestic product | Single threshold | 50.00 | 0.06 | 42.79 | 50.78 | 74.92 | 59753.37 | [57811.87,62474.79] |
|  | Double threshold | 40.12 | 0.17 | 62.79 | 83.91 | 119.48 | 59753.37 | [57811.87,62474.79] |
|  |  |  |  |  |  |  | 507.46 | [441.36,701.03] |

Note: This table shows the statistics of 31 provinces, autonomous regions, and municipalities directly under the central government in China.

**Extended Data Table 4. Threshold model results**

| **Threshold variable** | **Threshold effect** | **F value** | **Prob.** | **critical value** | | | **Threshold** | **95% confidence interval** |
| --- | --- | --- | --- | --- | --- | --- | --- | --- |
|  |  |  |  | **10%** | **5%** | **1%** |  |  |
| Per capita water resources | Single threshold | 79.38 | 0.03 | 42.29 | 63.18 | 85.26 | 3892.69 |  |
|  | Double threshold | 103.22 | 0.12 | 114.18 | 163.06 | 252.11 | 3892.69 |  |
|  |  |  |  |  |  |  | 1488.60 | [1484.66,1632.25] |
| Number of rural labor force | Single threshold | 35.00 | 0.15 | 39.63 | 45.97 | 55.55 | 1827.40 | [1810.15,1863.76] |
|  | Double threshold | 23.63 | 0.26 | 35.65 | 43.06 | 71.45 | 1827.40 | [1810.15,1856.60] |
|  |  |  |  |  |  |  | 876.27 | [858.93,881.88] |
| Disposable income | Single threshold | 38.59 | 0.04 | 28.13 | 35.22 | 52.12 | 21125.00 | [20706.90,21191.64] |
|  | Double threshold | 13.56 | 0.37 | 33.10 | 40.96 | 64.80 | 21125.00 | [20706.90,21191.64] |
|  |  |  |  |  |  |  | 5624.00 | [5082.70,5719.40] |
| Government attention | Single threshold | 24.11 | 0.12 | 25.12 | 28.52 | 40.69 | 6.26 | [6.21,6.33] |
|  | Double threshold | 11.57 | 0.47 | 25.71 | 33.01 | 53.68 | 6.26 | [6.21,6.33] |
|  |  |  |  |  |  |  | 14.52 | [14.09,14.60] |
| Foreign trade dependence | Single threshold | 44.33 | 0.02 | 27.66 | 32.18 | 47.10 | 0.31 | [0.30,0.31] |
|  | Double threshold | 23.11 | 0.14 | 25.44 | 28.43 | 45.53 | 0.31 | [0.30,0.31] |
|  |  |  |  |  |  |  | 1.50 | [1.42,1.50] |
| industrial structure | Single threshold | 44.22 | 0.03 | 37.22 | 42.15 | 49.37 | 0.61 | [0.48,0.65] |
|  | Double threshold | 23.19 | 0.30 | 59.48 | 80.83 | 127.94 | 0.51 | [0.41,0.53] |
|  |  |  |  |  |  |  | 12.81 | [12.55,13.02] |
| Gross domestic product | Single threshold | 50.00 | 0.06 | 42.79 | 50.78 | 74.92 | 59753.37 | [57811.87,62474.79] |
|  | Double threshold | 40.12 | 0.17 | 62.79 | 83.91 | 119.48 | 59753.37 | [57811.87,62474.79] |
|  |  |  |  |  |  |  | 507.46 | [441.36,701.03] |
